# The role of β-catenin in the initiation and metastasis of TA2 mice spontaneous breast cancer: Erratum

**DOI:** 10.7150/jca.103238

**Published:** 2024-09-12

**Authors:** Dan Zhang, Fei Fei, Shuyuan Li, Yongjie Zhao, Zhengduo Yang, Jie Qu, Xipeng Zhang, Yu Yin, Shiwu Zhang

**Affiliations:** 1 Department of Pathology, Tianjin Union Medical Center, Tianjin, 300121, China; 2 Nankai University School of Medicine, Nankai University, Tianjin, 300071, China; 3 Department of Colorectal Surgery, Tianjin Union Medical Center, Tianjin, 300121, China; 4 Department of General Surgery, Tianjin Union Medical Center, Tianjin 300121, China; 5 Department of Pathology, Anhui Medical University, Hefei 230032, China

In the original version of our article, there was an error in Fig 3. Specifically, the representative images of "Negative control cells" (Figure 3B a)" and siRNA control" (Figure 3B b) are incorrect, the presence of bands in the IgG group of the negative control in Figure 3F is incorrect. The correct image is provided below. This correction will not affect the results and conclusions. The authors apologize for any inconvenience this may have caused.

## Figures and Tables

**Figure 3:**
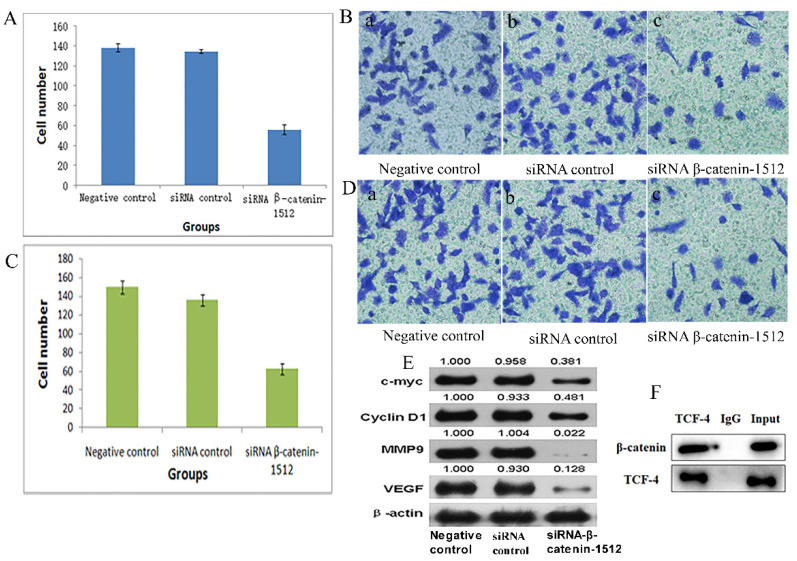
Low-expression of β-catenin inhibited the migration and invasion capability of MA-89 cell. A. Histogram shows the number of invading MA-891 cells with the negative control, siRNA control and siRNA β-catenin-1512 treatments. B. Results of Transwell experiments with MA-891 showing that siRNA β-catenin-1512 inhibits cell invasiveness. a) Negative control, b) siRNA control, c) siRNA β-catenin-1512. C. Histogram shows the number of migrating MA-891 cells with the negative control, siRNA control and siRNA β-catenin-1512 treatments. D. The number of migrations of MA-891 cells treated with siRNA β-catenin-1512 was lower than that found in the other two groups. a) Negative control, b) siRNA control, c) siRNA β-catenin-1512. E. The expression levels of C-myc, CyclinD1, MMP-9, and VEGF as determined by western blot analysis. F. Interaction between β-catenin and TCF-4 in MA-891 cells using Co-IP.

